# Progress and Perspectives Beyond Traditional RAFT Polymerization

**DOI:** 10.1002/advs.202001656

**Published:** 2020-08-26

**Authors:** Mitchell D. Nothling, Qiang Fu, Amin Reyhani, Stephanie Allison‐Logan, Kenward Jung, Jian Zhu, Masami Kamigaito, Cyrille Boyer, Greg G. Qiao

**Affiliations:** ^1^ Polymer Science Group Department of Chemical Engineering The University of Melbourne Parkville VIC 3010 Australia; ^2^ Centre for Technology in Water and Wastewater Treatment (CTWW) School of Civil and Environmental Engineering University of Technology Sydney Ultimo NSW 2007 Australia; ^3^ Centre for Advanced Macromolecular Design (CAMD) and Australian Centre for NanoMedicine (ACN) School of Chemical Engineering UNWS Sydney NSW 2052 Australia; ^4^ College of Chemistry Chemical Engineering and Material Science Department of Polymer Science and Engineering Soochow University Suzhou 215123 China; ^5^ Department of Molecular and Macromolecular Chemistry Graduate School of Engineering Nagoya University Furo‐cho, Chikusa‐ku Nagoya 464‐8603 Japan

**Keywords:** controlled/living polymerization, photochemistry, polymer structures, reversible addition‐fragmentation chain transfer (RAFT), spatiotemporal regulation

## Abstract

The development of advanced materials based on well‐defined polymeric architectures is proving to be a highly prosperous research direction across both industry and academia. Controlled radical polymerization techniques are receiving unprecedented attention, with reversible‐deactivation chain growth procedures now routinely leveraged to prepare exquisitely precise polymer products. Reversible addition‐fragmentation chain transfer (RAFT) polymerization is a powerful protocol within this domain, where the unique chemistry of thiocarbonylthio (TCT) compounds can be harnessed to control radical chain growth of vinyl polymers. With the intense recent focus on RAFT, new strategies for initiation and external control have emerged that are paving the way for preparing well‐defined polymers for demanding applications. In this work, the cutting‐edge innovations in RAFT that are opening up this technique to a broader suite of materials researchers are explored. Emerging strategies for activating TCTs are surveyed, which are providing access into traditionally challenging environments for reversible‐deactivation radical polymerization. The latest advances and future perspectives in applying RAFT‐derived polymers are also shared, with the goal to convey the rich potential of RAFT for an ever‐expanding range of high‐performance applications.

## Graphical abstract

Preparing precision vinyl polymers using reversible addition‐fragmentation chain transfer (RAFT) polymerization is a powerful tool in modern materials design. Unprecedented recent focus on RAFT has revealed new strategies for activation and spatio‐temporal control, eclipsing the role of traditional radical initiators. Herein, the latest advances in RAFT initiation are outlined and the challenging reaction settings and innovative applications now within reach are revealed.

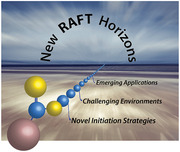

## 1 Introduction

There has been explosive interest and exploration in reversible‐deactivation radical polymerization (RDRP) for precision polymer synthesis over recent years. In particular, since the development of reversible addition‐fragmentation chain transfer (RAFT) by CSIRO researchers in 1998,^[^ ^1^ ^]^ the application of thiocarbonylthio (TCT) compounds for mediating radical polymerizations has occupied the forefront of precision polymer development across both industry and academia.^[^ ^2^, ^3^ ^]^ By facilitating degenerate chain transfer of propagating radical polymers to TCTs, the RAFT process provides a powerful living handle to regulate chain growth and to predetermine a polymer's physical and chemical characteristics. Driven by the versatility and ease of application of RAFT, as well as the widespread commercial availability of TCTs, a myriad of materials have been realized for varied applications, ranging from paints and engineering materials to healthcare and antibacterial coatings.^[^ ^4^ ^]^

Since our last perspective in 2016,^[^ ^5^ ^]^ the continued study of RAFT has accelerated into new, more challenging environments. Traditional radical activation via thermal initiators has been eclipsed by approaches that are more tailorable, specific, and compatible with external regulation. Such advances are expanding the value of RAFT to a broader scope of materials researchers.^[^ ^6^ ^]^ RAFT is now possible in settings where traditional radical polymerizations have seldom been applied, including aerobic and biological environments, in ultralow volumes and in high‐throughput or continuous flow processes. This progress has seen RAFT make impact in a range of advanced applications, yielding materials with unprecedented levels of structural detail, stimuli responsiveness, and biomedical relevance. Polymers with perfectly defined sequences, predefined ultrahigh molecular weights, targeted and tunable secondary/tertiary structures, and with complex interfaces to biological systems are now routinely prepared.

Challenges still remain in the scaling and translation of these techniques into industrial settings, as well as fully exploiting the mechanistic intricacies of RAFT to maximize control and versatility. In this Research News article, we will give insight into the most recent, cutting‐edge explorations of RAFT for preparing well‐defined polymeric architectures. An outline of the latest techniques for initiating and externally regulating RAFT is provided, which are supplanting the role of the traditional thermal radical initiator. Emerging innovative applications and potential new avenues for exploration are shared, with a goal to convey the exciting new directions available with RAFT to both seasoned researchers and those new to the field.

## 2 Nontraditional Activation of TCTs

In contrast to the reversible termination mechanism underpinning RDRP techniques like atom transfer radical polymerization (ATRP)^[^ ^7^, ^8^ ^]^ and nitroxide‐mediated polymerization (NMP),^[^ ^9^, ^10^ ^]^ RAFT mediates chain growth of vinyl polymers through degenerate chain transfer of a TCT to propagating (macro)radicals. RAFT therefore requires the continuous addition of (re)initiating radicals to compensate for unavoidable termination events and thereby sustain polymer chain growth. The original and still most widely explored approach to RAFT initiation is through thermally labile radical initiators, such as 2,2′‐azobisisobutyronitrile (AIBN).^[^ ^11^ ^]^ While well‐studied and easily applied, such exogenous initiators are inherently hazardous, require elevated temperatures, provide limited scope for temporal or spatial regulation, and include initiator‐derived residues into the polymer products.

**Figure 1:**
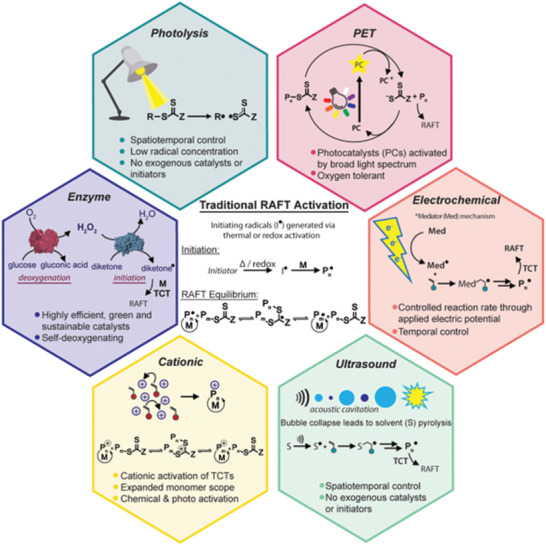
Emerging methods for activating RAFT polymerization. The traditional route of RAFT activation by adding exogenous, thermal‐ or redox‐labile reagents is being supplanted by novel activation strategies that can increase livingness and provide scope for external regulation.

Endogenous activation through radical genesis derived directly from TCT compounds provides an alternative, nontraditional route to initiate polymerization (**Figure** **1**). (In)direct activation of TCTs via an external stimulus is distinguished by an absence of exogenous radicals, whereby polymerization is promoted by radicals derived from the RAFT agent itself, with control maintained by chain transfer to dormant species as per the RAFT mechanism.^[^ ^12^, ^13^, ^14^, ^15^, ^16^, ^17^ ^]^ Much work has been directed at the photoactivation of TCTs for externally initiating RAFT, either through direct activation of the TCT itself (the so‐called photoiniferter method) or indirectly through energy transfer via a photoactivated chromophore.

### 2.1 Photoactivation of TCTs

#### 2.1.1 Direct Photoactivation (Photoiniferter)

The direct irradiation of trithiocarbonates with blue light (*λ* ≈ 450 nm), which corresponds to the n to π* transition, facilitates the cleavage of secondary C—S bonds to generate an R‐group (macro)radical, as independently demonstrated by the Qiao and Boyer groups.^[^ ^14^, ^18^ ^]^ By adjusting the TCT structure with different R‐group substitutions, the selective photoactivation of TCTs can be achieved under different wavelengths of light. For example, TCTs possessing tertiary R‐group fragments can be selectively photocleaved under green light (*λ* ≈ 530 nm).^[^ ^14^ ^]^ Matyjaszewski and co‐workers harnessed this effect in the preparation of comb‐like and bottlebrush polymers in a single reaction mixture.^[^ ^19^ ^]^

The work of Poly showed that both blue and green LED irradiation could be used for the RAFT polymerization of butyl acrylate (BA) after swapping out the trithiocarbonate RAFT agent for a dithiocarbamate.^[^ ^20^ ^]^ Alternatively, the light source could also be extended from blue light to near‐infrared light (*λ* ≈ 980 nm) by adding upconversion nanoparticles into the polymerization system, which serve as an internal light source for activating a xanthate RAFT agent.^[^ ^21^ ^]^ Importantly, tuning the intensity of light irradiation in these systems can regulate the polymerization rate, where increasing the LED power from 6 to 208 W may result in a drastic reduction in polymerization time from 12 h to 11 min.^[^ ^22^ ^]^ Further control over the polymerization can also be provided by tuning the reaction temperature during photoactivation, as shown by Zhu and co‐workers.^[^ ^23^ ^]^ In this work, the tacticity of vinyl acetate photopolymerization could be controlled by adjusting the reaction temperature, where hydrogen bonding between solvent and monomer changes with temperature to provide stereoregularity to the propagating chain end. The triple role of the TCT in photoiniferter RAFT provides for a greatly simplified reaction mixture, which should benefit industrial translation by reducing the cost and complexity of RAFT‐derived materials.

#### 2.1.2 Indirect Photoactivation

Photoactivation of TCTs can also be achieved indirectly, where excited photoredox catalysts are employed to activate so‐called photoinduced electron/energy transfer (PET)‐RAFT polymerization.^[^ ^15^, ^16^, ^24^, ^25^, ^26^, ^27^, ^28^ ^]^ The superior photophysical characteristics of many photocatalysts compared with TCTs enable faster polymerizations that proceed under broader wavelengths of visible and NIR irradiation. These extended wavelengths, particularly those in the NIR region, are especially attractive as they not only provide enhanced material penetration^[^ ^29^, ^30^, ^31^ ^]^ but also can further widen the absorption gap between common photoactive species present in the reaction, avoiding unintended side reactions.^[^ ^32^ ^]^

The PET‐RAFT mechanism has been purported to proceed via electron or energy transfer depending on the catalyst type; mechanisms proceeding via the former have been further delineated into oxidative and reductive quenching pathways.^[^ ^33^, ^34^, ^35^ ^]^ Moreover, dependent on the wavelength used, the concomitant direct activation of the TCT cannot be ruled out.

An interesting recent case of catalyst selectivity was reported by Boyer and co‐workers who discovered that the organic catalyst pheophorbide a (PheoA) specifically activated a dithiobenzoate RAFT agent.^[^ ^36^ ^]^ This specificity was exploited to successfully polymerize a trithiocarbonate‐functionalized methacrylate under red light. These pendant trithiocarbonates were then specifically activated by zinc tetraphenylporphyrin (ZnTPP) under green light to produce graft copolymers. As the library of photocatalysts compatible with PET‐RAFT continues to grow, there is potential for the discovery of further novel capabilities and enhanced polymer features. In particular, the development of heterogeneous catalysts is receiving increasing attention, as it provides both easier separation and potential for recycling.^[^ ^37^, ^38^, ^39^, ^40^, ^41^, ^42^, ^43^, ^44^ ^]^ In parallel, computational methods are showing promise for the rational optimization of existing photocatalysts and the incorporation of new synthetic capabilities.^[^ ^45^, ^46^ ^]^

In addition to PET‐RAFT, photoinitiators capable of directly forming radicals via homolysis under light have continued to receive attention for activating RAFT. While less sophisticated than the PET and photoiniferter systems discussed so far, photoinitiation offers access to external regulation and mild reaction conditions and has even been shown using vinyl ketone monomers as intrinsic photoinitiators.^[^ ^47^, ^48^ ^]^ As with all photoactivated chemistry, limitations are imposed by the need for a suitable, uniform light source and transparent reaction vessels. Furthermore, non‐uniform irradiation of reaction mixtures, especially at high reagent concentrations, can impact reproducibility and hinder scale‐up. Recent work with chemiluminescence as a light source for activating photoinitiated RAFT has offset some of these limitations, where in situ light generation can be used to increase irradiation uniformity.^[^ ^49^, ^50^ ^]^

### 2.2 Electro‐RAFT

While photochemical routes to activate TCTs have received the largest focus by researchers, electrochemical methods have recently emerged as a potential new route for RAFT initiation. The goal of such techniques is the direct electroreduction of TCT compounds under an applied electric field to furnish a (macro)radical species for initiating chain growth. Although such an eRAFT approach should afford similar benefits to photoactivation, including the tunability of the external stimuli for rate and temporal control, the direct single electron reduction of TCTs via an applied electric current has so far proven insurmountable. Instead, electroreduction appears to irreversibly cleave TCTs at the weak C—S bond to generate anionic fragments, which undergo a variety of side reactions without producing any long‐lived radical species.^[^ ^51^, ^52^ ^]^ To circumvent this effect, electroactive mediators have shown promise for generating radicals in the presence of a reducing electric field, which can initiate and sustain eRAFT indirectly.^[^ ^53^ ^]^ In addition, a mediated approach using electroactivated oxidizing agents has also been successfully applied by the Fors and Yan groups for indirect TCT oxidation, leading to well‐controlled cationic RAFT polymerization (vide infra) with excellent temporal regulation.^[^ ^54^, ^55^ ^]^ The use of redox active mediating compounds appears essential for interfacing RAFT polymerization with electrochemical initiation and control.

### 2.3 Sono‐RAFT

Another recent alternative for externally regulated RAFT polymerization is through the application of ultrasound irradiation to generate radicals directly from the reaction solvent. Acoustic cavitation of the polymerization solvent under the stimulus of high frequency (>≈400 kHz) ultrasound causes homolysis of solvent molecules to create radicals. While distinct from the novel TCT‐derived radical initiation strategies discussed so far, sonoactivation provides many of the same benefits, including removal of an exogenous radial initiator and provision of external regulation of polymerization progress. Although the chemical effects of ultrasound have been recognized in free radical polymerization for some time,^[^ ^56^ ^]^ Qiao and co‐workers showed the first example of so‐called sono‐RAFT, highlighting the rapid polymerization rate, temporal regulation, and outstanding control of product structure afforded by this technique.^[^ ^57^ ^]^ Sonochemical initiation has since proven effective in a range of aqueous and organic systems where •OH radicals or various carbon‐centered radicals constitute the predominant initiating species, respectively.^[^ ^58^ ^]^ Solvents exhibiting both low vapor pressure and low room‐temperature viscosity (such as dimethylformamide and dimethylacetamide) seemed best suited for sono‐derived radical generation and organic sono‐RAFT. More recently, sono‐RAFT has been applied for the preparation of controlled polymers with targeted self‐assembly properties, yielding thermoresponsive nanogels when applied to dispersed media.^[^ ^59^ ^]^ Challenges with the sonomechanical cleavage of polymers at high molecular weights and reduced radical production with increasing reaction viscosity during sono‐RAFT require further study. Furthermore, the ultrasonic activation of polymerization coupled with mechano‐, thermo‐, or photochemical control may prove fruitful new directions for research.

### 2.4 Cationic (Photo‐)RAFT

The versatility of TCT compounds for activation by propagating radical chain growth polymers was extended in the work of Kamigaito, who showed that these materials are also responsive to cationic activation.^[^ ^60^ ^]^ By providing for degenerate addition‐fragmentation of cationic species, RAFT has been expanded to afford control over the cationic polymerization of a broader suite of monomer types, including vinyl ethers and styrenes.^[^ ^61^ ^]^ Photoinduced oxidation of TCTs has emerged as a dominant route for generating cationic propagating species and initiating polymerization.^[^ ^62^ ^]^ Organic pyrylium and iridium photocatalysts have both proven highly effective in the presence of trithiocarbonates and dithiocarbamates under visible light for controlling cationic polymerization of various vinyl ethers.^[^ ^62^, ^63^ ^]^ As with radical photoRAFT, temporal control is readily achieved by switching ON/OFF light irradiation, which can be further enhanced by selection of a photocatalyst with greater stability (i.e., iridium complexes).^[^ ^64^ ^]^

An interesting application to emerge from this technology has been the ability to combine orthogonal cationic and radical RAFT polymerizations. Combining these mechanistically distinct transformations enables the synthesis of unique block and star polymers consisting of cationically and radically polymerizable monomers, which cannot be accessed by a single polymerization in isolation.^[^ ^65^, ^66^, ^67^, ^68^ ^]^ An extension of this strategy lies in the simultaneous application of both Lewis acid and thermal radical initiators to induce an interconvertible controlled cationic and radical polymerization in one pot.^[^ ^69^ ^]^ Under this strategy, complex multiblock copolymers of vinyl ethers and acrylates or vinyl acetate can be realized, with sequence distributions afforded by careful selection of an initiator capable of monomer kinetic resolution.^[^ ^69^, ^70^ ^]^ Latest developments have seen two separate RAFT agents employed that individually control the orthogonal cationic and radical chain transfer processes, leading to unprecedented bottlebrush copolymers.^[^ ^71^ ^]^ The exploration of interconvertible RAFT has also recently been elegantly expanded by the Fors group, where visible light irradiation has been employed to toggle cationic and radical RAFT activation in a single reaction mixture.^[^ ^72^ ^]^ Orthogonal photocatalysts are key to this approach, where external regulation of the wavelength of light can afford remarkable levels of complexity and control, furnishing complex, sequence‐defined copolymers.^[^ ^72^, ^73^ ^]^

## 3 RAFT in Unique Environments

**Figure 2:**
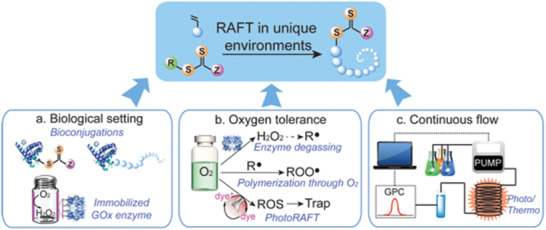
RAFT is now possible in reaction settings where traditional radical polymerization has seldom been applied. Such advances pave the way for RAFT to make impact in new and emerging applications, including aerobic and biological settings and in continuous flow processes.

The versatility and reliability of RAFT has seen it employed in remarkably diverse settings, from neat aqueous and organic solvents, to bulk and dispersed phases, from solid surfaces and across a wide range of temperatures. Building on this varied exploration of RAFT, recent research has expanded the technique into yet broader reaction environments where conventional free radical polymerization has seldom been applied (**Figure** **2**).

### 3.1 Biological Settings and Enzymatic Activation

The in situ synthesis of polymers in biological settings (e.g., culture media or bodily fluids under physiological conditions) is an exciting advance for preparing polymers with biomedical potential. However, the presence of diverse molecular species in biological media has traditionally precluded RDRP in such settings, where the biological milieu can interfere with reversible‐deactivation polymerization. Qiao and co‐workers described a well‐controlled RAFT polymerization in cell culture media and in whole sheep blood initiated entirely by biological reagents via the Fenton reaction.^[^ ^74^ ^]^ In this work, the hemoglobin in blood provides a source of iron ions, which combine with H_2_O_2_ delivered by the activity of glucose oxidase to generate hydroxyl radicals. The highly reactive Fenton hydroxyl radicals provide rapid polymerization rates and afforded the synthesis of ultrahigh molecular weight (UHMW) polymers, while not disturbing the integrity of the biomolecular components.^[^ ^75^ ^]^

In many applications, the covalent modification of biomolecules with polymers is desirable, with the goal of tuning their inherent physical and chemical properties to impart new qualities such as enhanced stability, stimuli‐responsiveness, and lower immunogenicity.^[^ ^76^, ^77^ ^]^ The mild conditions provided by visible light mediated RAFT polymerizations have been attractive for the preparation of bioconjugates. Hawker and co‐workers highlighted the biocompatibility of the PET‐RAFT process by growing controlled polymers directly from the surface of live cells, thereby modifying cellular phenotype without disturbing the cell viability.^[^ ^78^ ^]^ Further demonstration of the power of PET‐RAFT in biological settings is confirmed by the successful polymerization of peptide‐based macromonomers, as well as grafting from proteins and DNA.^[^ ^79^, ^80^, ^81^ ^]^ In the future, leveraging RAFT in the development of functional bioconjugates will be a great source of future innovation to support nascent biomedical applications, such as personalized medicine.

The benefits of mild, aqueous reaction conditions, high specificity, and unrivalled rate enhancement associated with biocatalysis have seen enzymes increasingly exploited for green materials synthesis.^[^ ^82^, ^83^ ^]^ Recently, enzyme catalysis has been applied in RAFT polymerization to fulfill two key roles: to remove dissolved oxygen in aqueous reaction mixtures, and to facilitate a flux of initiating radicals. The Stevens group introduced the widely explored and commercially available oxidoreductase glucose oxidase (GOx) to purge molecular oxygen from RAFT reaction mixtures.^[^ ^84^, ^85^ ^]^ The outstanding efficiency of GOx afforded effective O_2_ removal with nanomolar enzyme concentrations, allowing a high‐throughput RAFT to proceed in open air and low (≈10 µL) reaction volumes. The resilience of GOx was entertainingly illustrated by Rowan and co‐workers, where GOx deoxygenation preceded successful RAFT polymerization in a comprehensive range of alcoholic beverages.^[^ ^86^ ^]^ In these studies, the enzyme component is employed solely for deoxygenation, with radical initiation provided by traditional azo‐thermal initiators. While this enzyme‐decoupled initiation strategy affords high chain‐end fidelity by controlling the initiator to TCT ratio, the build‐up of deoxygenation product H_2_O_2_ may result in undesired oxidative side‐ reactions.

To circumvent such issues, An and co‐workers designed an enzymatic cascade, where the H_2_O_2_ from pyranose oxidase deoxygenation could be subsequently reduced by a second enzyme, horseradish peroxidase, yielding radicals through the accompanied oxidation of acetylacetone.^[^ ^87^ ^]^ This entirely enzymatic route to activate RAFT furnished multiblock copolymers and ultrahigh molecular weight (>10^6^ g mol^−1^) homopolymers without additional precautions for oxygen exclusion. Further study has consolidated the effectiveness of enzyme‐coupled initiation,^[^ ^88^ ^]^ and horseradish peroxidase in general,^[^ ^89^, ^90^ ^]^ for undertaking green RAFT polymerization. The common issue of limited enzyme stability in diverse reaction conditions may confine these approaches to aqueous, ambient polymerizations with hydrophilic monomers. To increase the industrial relevance of these strategies, Qiao and co‐workers explored enzyme immobilization to improve the stability and reusability of the enzyme catalysts.^[^ ^91^ ^]^ An innovative interfacing of oxidoreductase enzymes with photocatalysis has also emerged recently, where the photoexcitation of the enzyme's flavin cofactor can be directly harnessed to induce PET‐RAFT.^[^ ^92^ ^]^

### 3.2 Oxygen‐Tolerant RAFT

The issue of interference by molecular oxygen (O_2_) in radical polymerizations is a common dilemma that has compelled the design of a multitude of techniques for removing or reacting with dissolved oxygen.^[^ ^93^ ^]^ Polymerizing‐through oxygen represents a straightforward approach for O_2_‐tolerant RAFT, where traditional thermal‐ or redox‐generated radicals can sacrificially consume oxygen prior to initiating polymerization. Though such approaches are attractive for their operational simplicity, the frequent constraints of low polymer chain lengths and high required initiator and monomer concentrations limit their versatility.^[^ ^93^ ^]^ Recently, the singlet oxygen generation capabilities inherent to many photoredox catalysts have provided an accessible and effective approach to performing photopolymerizations without the need for deoxygenation processes.^[^ ^93^, ^94^, ^95^, ^96^, ^97^ ^]^ PET from common photocatalysts to molecular oxygen via triplet–triplet annihilation can drive the conversion of dissolved molecular oxygen into a singlet excited state. When paired with suitable singlet oxygen quenchers, such as anthracene or even the common organic solvent dimethylsulfoxide (DMSO), reactive oxygen present in the reaction mixture is gradually sequestered.^[^ ^98^, ^99^, ^100^, ^101^ ^]^ Alternatively, dissolved oxygen may also be converted into a source of radicals. Dye/ascorbic acid initiation systems provide oxygen tolerance through the photochemical conversion of oxygen into peroxide, which is further reduced to hydroxyl radicals in the presence of ascorbic acid, which can activate polymerization.^[^ ^102^, ^103^ ^]^ Zinc octaethyl tetraphenyl porphyrin (ZnOETPP) was recently reported as a photocatalyst for PET‐RAFT, which possessed the peculiar capability of utilizing oxygen as a cocatalyst in conjunction with triethylamine.^[^ ^104^ ^]^ Surprisingly, no polymerization was observed in inert gases such as nitrogen and carbon dioxide, whereas polymerization could proceed unhindered in air, and even be accelerated in a pure oxygen environment.

The ability to conduct polymerizations free from the limitations of deoxygenation processes has opened new applications that were previously inaccessible. For instance, the ability to polymerize at low to ultralow (<10 µL) volumes has allowed the transition of this technology to the benchtop for combinatorial and high‐throughput chemistry. In addition to these applications, oxygen tolerance has also significantly simplified the reaction setup for performing surface‐initiated polymerizations,^[^ ^105^, ^106^ ^]^ and for the translation of RAFT into 3D printing.^[^ ^107^, ^108^ ^]^ Complimenting the observed improvements in interlayer bonding, the application of RAFT holds the promise of finer control over the nano/microstructure of printed materials and the programming of new functions and stimuli‐responsivity.

### 3.3 Continuous Flow

The translation of precision polymer synthesis into continuous flow can provide an array of processing benefits, including improved heat transfer, enhanced mixing, and online adjustment capabilities.^[^ ^109^ ^]^ In particular, photoRAFT is well suited to a continuous flow regime due to the short optical path lengths in photoflow reactors, resulting in negligible intensity gradient compared with conventional batch photopolymerization systems.^[^ ^110^ ^]^ Leveraging this advantage, Junkers and Wenn conducted a series of RAFT photopolymerizations of butyl acrylate using various photocatalysts under UV irradiation.^[^ ^111^ ^]^ Interestingly, the authors observed fast monomer conversion in the first few minutes of flow, followed by an almost linear evolution of ln(conv.). They attributed this effect to the rapid initial consumption of photocatalyst preceding a classical photoiniferter polymerization. The Junkers group followed up this study employing blue light to induce photoiniferter RAFT polymerizations of various methacrylates.^[^ ^112^ ^]^ By prudent selection of RAFT agent, polymethacrylates were obtained with controlled molecular weights, narrow dispersity (*Đ* < 1.2) and high chain‐end fidelity.

Corrigan et al. introduced oxygen tolerance into a flow process by employing trace amounts of zinc tetraphenylporphyrin (ZnTPP) as PET‐RAFT photocatalyst for singlet oxygen generation.^[^ ^98^ ^]^ After the removal of oxygen with DMSO, polymers were then continuously produced with controlled molecular weights and narrow dispersity via RAFT.^[^ ^113^ ^]^ Such a flow system was also employed to fabricate polymeric nanoparticles of tunable morphology in the presence of a polyethylene glycol (PEG)‐based macroRAFT agent and hydroxypropyl methacrylate (HPMA) via aqueous PET‐RAFT polymerization‐induced self‐assembly (PISA).^[^ ^114^ ^]^ Extending the development of block copolymers under flow, Perrier and co‐workers designed a looped flow system that could afford the near quantitative conversion of monomers via loop circulation. By uniting this system with sequenced monomer injection, well‐defined hexablock copolymer products with narrow dispersity could be furnished in a continuous fashion.^[^ ^115^ ^]^ Looking forward, issues with processing the increasingly viscous reaction solution during continuous flow polymerization may pose some constraints on feasibility and industrial translation of this technique. However, working under dilute or dispersed conditions, and the increased understanding of alternative process designs continue to offset such issues, ensuring sustained innovation in this field.^[^ ^116^ ^]^

## 4 Advanced and Emerging Applications of RAFT

**Figure 3:**
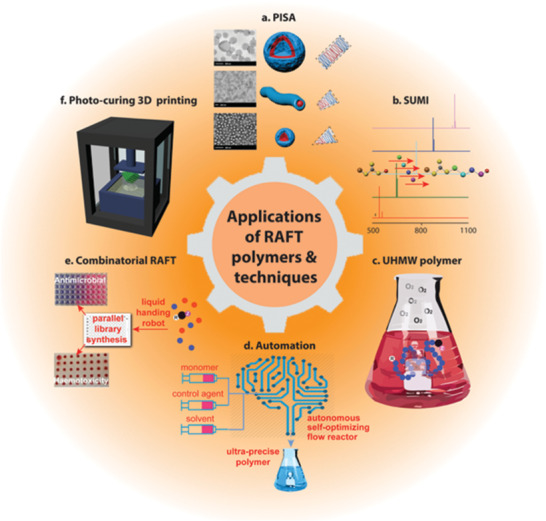
Innovative new applications of RAFT polymerization. a) Polymeric micelle, rod and vesicle nanoparticle morphologies synthesized via polymerization‐induced self‐assembly (PISA).^[^ ^117^ ^]^ Adapted with permission.^118^, ^119^ Copyright 2011, American Chemical Society; and Copyright 2013, American Chemical Society, respectively. b) Light‐induced RAFT single‐unit monomer insertion (SUMI) technique for the preparation of sequence‐controlled oligomers.^[^ ^120^ ^]^ c) Enzyme/(nanozyme) facilitated RAFT polymerization to prepare UHMW polymers in air^[^ ^87^ ^]^ also achieved efficiently using photoiniferter RAFT.^[^ ^121^ ^]^ d) Autonomous self‐optimizing flow reactors afford online optimization of RAFT.^[^ ^122^ ^]^ e) Combinatorial discovery of antimicrobial copolymers via PET‐RAFT polymerization. f) The use of PET‐RAFT to develop visible‐light‐mediated photocuring techniques for application in 3D printing.^[^ ^123^ ^]^

The broad uptake of RAFT in the polymer and materials science communities is increasingly paving the way for new and expanded applications of RAFT‐derived polymers. Hereto unforeseen capabilities of controlled vinyl polymers have been discovered and expanded, with RAFT playing a key role in unlocking access to these advanced materials (**Figure** **3**).

### 4.1 Polymerization‐Induced Self‐Assembly

PISA has been a revelation for the facile preparation of anisotropic polymeric nanoparticles with tunable morphology.^[^ ^117^, ^124^, ^125^, ^126^, ^127^, ^128^, ^129^ ^]^ Compared to conventional self‐assembly techniques that are conducted at high dilution (<1% (w/w)), the in situ chain extension of a solvophilic macroRAFT agent under dispersion or emulsion conditions facilitates reproducible syntheses of nanoparticles at significantly higher concentrations (10–50% (w/w)). Building on the robustness of conventional, thermally initiated RAFT, there has been recent focus on the photoregulation of PISA.^[^ ^130^, ^131^ ^]^ Tan et al. demonstrated the robustness and speed of photoPISA initiated using visible light photoinitiators.^[^ ^132^, ^133^ ^]^ In an interesting report, sunlight was used as the irradiation source for the successful in situ encapsulation of fluorescently labeled bovine serum albumin.^[^ ^133^ ^]^ The direct activation of RAFT agents has been demonstrated to be a robust alternative facilitating additive free synthesis of various higher‐order morphologies.^[^ ^134^, ^135^ ^]^ Notably, for a given formulation, differences in final morphologies were observed when utilizing either higher energy wavelengths or higher intensities, which was attributed to a loss of end group fidelity. In all approaches, visual monitoring in conjunction with temporal control, implemented by simply switching the light ON/OFF, enables facile isolation of morphologies in contrast to thermally mediated processes. Taking advantage of the mild room temperature conditions, Gibson, O'Reilly and co‐workers reported the one‐pot in situ encapsulation of l‐asparaginase into the lumen of vesicles via photoPISA as an alternative to the covalent modification of proteins.^[^ ^136^ ^]^ Critically, the vesicles exhibited size‐selective permeability, which simultaneously conferred protection from proteases and antibody recognition while providing access to the asparagine substrate.

### 4.2 Single‐Unit Monomer Insertion (SUMI)

Inspired by the precision of natural biopolymers, the synthesis of polymers with uniform molecular weight and precisely defined monomer sequence has long been regarded as a holy grail of polymer chemistry.^[^ ^137^, ^138^, ^139^, ^140^ ^]^ The technique of SUMI has emerged as a highly promising strategy for the preparation of sequence‐defined polymers.^[^ ^120^ ^]^ Notably, SUMI via PET‐RAFT exploits the orthogonality of photocatalyst activation under different wavelengths of light to selectively cleave the C—S bonds of target TCT compounds. In tandem with monomers possessing slow polymerization rates or an inability to homopolymerize, the iterative insertion of vinyl monomers can be achieved without the need for a solid support or template.^[^ ^141^, ^142^, ^143^, ^144^, ^145^ ^]^ In work by Boyer and co‐workers, a styrenic monomer was first inserted into a trithiocarbonate species under green light irradiation, whereby selective activation of the tertiary R‐group, and not the secondary C—S bond of the product, ensured the absence of polymerization. Subsequently, staged irradiation of orthogonal photoredox catalysts Ir(ppy)_3_ and ZnTPP under blue and red light, respectively, afforded the sequential insertion of a maleimide monomer, then vinyl acetate or limonene with great precision. More recently, indene and maleimide monomers less capable of homopolymerization were selected for an alternating single insertion strategy.^[^ ^143^ ^]^ The electron‐accepting qualities of maleimides and electron‐donating qualities of indene necessitated an electron‐donating or accepting R‐group, respectively. Interestingly, the insertions were found to be *trans* only, resulting in oligomers that were both sequence‐defined and stereospecific.

### 4.3 Ultrahigh Molecular Weight

The fabrication of controlled UHMW polymers (defined by *M*_n_ > 10^6^ g mol^−1^) is a promising technique for preparing materials with outstanding mechanical properties.^[^ ^146^ ^]^ Key to the preparation of UHMW polymers is the application of a highly living propagation process, where a low instantaneous radical concentration is maintained throughout the polymerization so that bimolecular termination events can be minimized and chain growth can continue to beyond 10,000 repeat units. Sumerlin and co‐workers pioneered the synthesis of controlled UHMW acrylamido polymers by exploiting the high livingness of photoRAFT, which was recently extended to low *k*_p_ monomers in organic solvents.^[^ ^121^, ^147^ ^]^ Employing a similar photoiniferter technique, Qiao and co‐workers synthesized UHMW p(DMA) star polymers with 4 and 21 arms using a core‐first approach.^[^ ^148^ ^]^ Using a TCT‐functionalized *β*‐cyclodextrin core, star polymers in excess of 20 × 10^6^ g mol^−1^ were reported. Enzyme‐mediated RAFT has also emerged as a suitable technique for preparing UHMW polymers, due in part to the low, continual radical flux that can be sustained by biocatalysis.^[^ ^149^, ^150^, ^151^ ^]^ Maximizing the highly living nature of the RAFT process through a deeper mechanistic understanding, optimized reaction conditions and consideration of kinetic constraints will form the basis of UHMW polymer synthesis for future high‐performance materials.

### 4.4 Automation, Combinatorial, and High‐Throughput RAFT

Combinatorial and high‐throughput methodologies using automated synthesizers is an attractive strategy for fast tracking reaction optimization and development of libraries for structure–activity screening.^[^ ^152^, ^153^, ^154^, ^155^ ^]^ In recent years, the ability to conduct RAFT without traditional deoxygenation protocols has facilitated a growing number of benchtop combinatorial and high‐throughput studies, which are particularly advantageous for generating polymers with specific biological interactions. Enzymatic degassing^[^ ^84^, ^156^ ^]^ and photochemical oxygen sequestration^[^ ^155^, ^157^, ^158^, ^159^, ^160^, ^161^, ^162^, ^163^, ^164^, ^165^, ^166^ ^]^ have afforded access to (ultra)low reaction volumes and finely controlled reagent conditions that are common prerequisites for high‐throughput translation.

Despite this, bottlenecking during the subsequent characterizations may be the limiting factor to conducting massively parallel RAFT polymerizations. To overcome these challenges, Boyer's group exploited the fluorescence emission shift of ZnTPP, which was strongly correlated to monomer conversion during PET‐RAFT, to enable high‐throughput online monitoring using a fluorescent plate reader.^[^ ^167^ ^]^ An exciting advance in automated RAFT was recently disclosed by the team of Junkers, where a continuous flow polymerization reactor was coupled to online size exclusion chromatography (SEC) analysis and a machine‐learning protocol for self‐optimization.^[^ ^168^ ^]^ Remarkably, the automated program was capable of continuous monitoring of the polymer products and could rapidly tune the reaction parameters (i.e., residence time, monomer concentration, and control agent/initiator concentration) to accurately produce targeted products with predetermined molecular weight and dispersity. While the specialized and expensive nature of many automated systems may be a barrier for general uptake by the polymer science community, applications in which high reproducibility and scale are paramount (e.g., the pharmaceutical industry) will greatly benefit from these nascent technologies.

## 5 Future Outlook

With the impressive depth and scale of recent work being conducted with RAFT, the future impact of this technique is rapidly expanding. Although the mechanistic minutiae of RAFT are now well‐established, opportunities still exist to leverage such details to maximize livingness and squeeze out yet higher product quality. For example, recent studies in dispersed media have highlighted the importance of understanding RAFT kinetics for decreasing termination rates, directly improving block copolymer products.^[^ ^169^, ^170^ ^]^ In recent years, control over the molecular weight distribution has received growing interest as it provides a pathway to manipulate the physical properties of polymeric materials. Fors’ and Boyer's groups have introduced different strategies to achieve such control.^[^ ^171^, ^172^ ^]^ More recent work by Anastasaki and co‐workers into tailoring polymer dispersity by mixing high‐ and low‐activity TCT compounds also holds considerable promise for tuning the bulk physical properties of RAFT‐derived polymers.^[^ ^173^ ^]^ Furthermore, the effect of instantaneous initiator‐derived radical concentrations on RAFT has received scant attention, which may reveal opportunities for improving traditional initiation systems.^[^ ^174^ ^]^ The ability to scale down polymerization volumes by leveraging rapid reaction rates and without degassing, complemented by machine learning reaction designs, will provide further development in programmable polymer structures with precision sequences. With great urgency, the social responsibility of polymer chemists to address the intensifying issue of plastic pollution must also be acknowledged. In this regard, progress with sustainable sources of vinyl monomers,^[^ ^175^, ^176^ ^]^ and building‐in degradability into vinyl polymers should take precedence.^[^ ^177^ ^]^ Parallel progress in machine learning and automated synthetic chemistry will also inevitably impact the study of RAFT. Such advances will afford the ability to predesign material properties in silico and to prepare and test massive arrays of precision polymers, well outside the capability of human experimentalists. With the recent expiration of the original RAFT patents, increased uptake of RAFT in industry may be anticipated and ongoing work to introduce RAFT into existing radical polymerization processes should prove highly beneficial.

## 6 Conclusion

While the exploration of RAFT has been ongoing for over a quarter of a century, the accelerating volume of recent work highlights the continued relevance of this technique for precision polymer synthesis. Advances in activation and spatio‐temporal regulation of RAFT are introducing a broader suite of researchers to the versatility of RAFT, with implications across an ever‐expanding range of industries. Access to challenging reaction environments and polymer architectures of extraordinary structural detail via RAFT is now well within reach. Furthermore, this Research News provides only a selection of the broad and diverse landscape that is currently being explored by RAFT researchers. Continued focus on industrial translation and drive to complete our understanding of the RAFT mechanism will underpin the realization of the full potential of this technique into the future.

## Conflict of Interest

The authors declare no conflict of interest.
